# Cross-Sectional Analysis of Paronychias in the National Electronic Injury Surveillance System 1999–2018

**DOI:** 10.1159/000525032

**Published:** 2022-06-08

**Authors:** Amar D. Desai, Yu Wang, Cajeton Clint Nadarajah, Shari R. Lipner

**Affiliations:** ^a^Rutgers New Jersey Medical School, Newark, New Jersey, USA; ^b^Department of Dermatology, Wake Forest University School of Medicine, Winston-Salem, North Carolina, USA; ^c^Stony Brook University, Stony Brook, New York, USA; ^d^Department of Dermatology, Weill Cornell Medicine, New York, New York, USA

**Keywords:** Paronychia, Nail infections, NEISS, Nails, Nail disease, Emergency department

## Abstract

**Introduction:**

Paronychia is the most common hand infection. Prior paronychia studies were limited by small patient numbers. We conducted a national-level analysis over two decades, analyzing demographics, etiologies, and trends in paronychia cases.

**Methods:**

We conducted a retrospective analysis of paronychia cases in the 1999–2018 National Electronic Injury Surveillance System database. Sex, race, age, and cause were recorded and compared using χ<sup>2</sup>, ANOVA, and *t* tests. Multivariable linear regression analysis assessed changes in age, weight, and sex over time.

**Results:**

We analyzed a total of 2,512 cases, with an average age of 27.6 ± 20.6 years, 45.5% females, and 25.6% white and 28.6% black patients. In multivariable linear regression, both age and weight significantly increased over time. Manicuring was the most common etiology (30.9%), increasing in incidence over time and with a higher frequency in adults (*p* < 0.0001) and females (*p* < 0.0001). There was a significant decrease in pediatric paronychia cases over time, particularly in 0- to 4-year-olds. Possible limitations include missed paronychia cases or additional non-paronychia cases due to improper coding, infrequent race reporting, and inability to analyze treatments or distinguish between paronychia subtypes.

**Conclusions:**

Paronychia cases were associated with increased age and weight over time with different presentations by age. Manicuring represents the largest growing paronychia etiology.

## Introduction

Paronychia is an infection or inflammation of the nail folds [1, 2]. Subtypes include acute, chronic, and chemotherapy-associated paronychias. Acute paronychia is typically due to a bacterial infection, while chronic paronychia is frequently caused by irritants [3, 4, 5]. The chemotherapy-associated paronychia is a unique paronychia subtype due to various chemotherapeutics, most commonly epidermal growth factor receptor inhibitors and taxanes [6, 7]. These distinct paronychia subtypes require different treatment strategies. Acute paronychias typically require drainage with or without antibiotics based on culture results [8, 9, 10, 11]. Chronic paronychias are best prevented by avoiding exposure to contact irritants and are managed with topical anti-inflammatories and/or broad-spectrum antifungal agents [12]. While there are no standardized treatments for chemotherapy-associated paronychias, there is evidence supporting avoidance of trauma, topical anti-inflammatories, oral tetracyclines, and 2% povidone-iodine solution for management [7, 13].

While paronychia in its acute form is the most common type of hand infection [14], single-institution studies have been limited by sample size, making it difficult to draw definitive conclusions about treatment strategies. Furthermore, few studies have investigated paronychia causes, with a dearth of information regarding proper preventative practices. Important racial, gender, and age-based recommendations have not been previously possible due to these small sample sizes and lack of race reporting.

Given the paucity of data regarding demographics and etiologies of paronychia cases, research on larger samples is critical to understand prevention, diagnosis, and management. In this study, we conducted a retrospective analysis of paronychia cases in the 1999–2018 National Electronic Injury Surveillance System (NEISS) multi-center database, representing the largest known national-level analysis of paronychias to date.

## Materials and Methods

A retrospective analysis of paronychia cases was performed using the NEISS database encompassing the time period December 1999 to December 2018. The NEISS is a nationally representative stratified probability sample of 66 hospitals having at least 6 beds and providing 24-h emergency services within the USA. Data for each paronychia case included a short summary narrative written by a NEISS medical abstractor. These summary narratives were written by representative emergency room personnel, which may not have included a board-certified dermatologist. Included cases were identified by utilizing the program language JavaScript to search for the keyword “paronychia” in the short narrative section of each case. NEISS is a publicly available database.

Clinical and demographic characteristics were obtained for each case including patient age, weight, race, affected digit/toe, etiology, and date of the recorded encounter. Etiology was coded based on the 2019 NEISS Coding Manual. The top ten causes by frequency were recorded, with all others recorded as “other etiologies”.

Univariable and multivariable analyses were performed using Microsoft Excel (Microsoft, Seattle, WA) and SAS Software (SAS Studio Release 3.8, Cary, NC, USA). Demographics were compared using a combination of χ^2^, ANOVA, and *t* tests. Multivariable linear regression analysis was performed to assess changes in age, weight, and sex over time. Statistical tests were run with a two-sided alpha-value of 0.05.

## Results

**Fig. 1:**
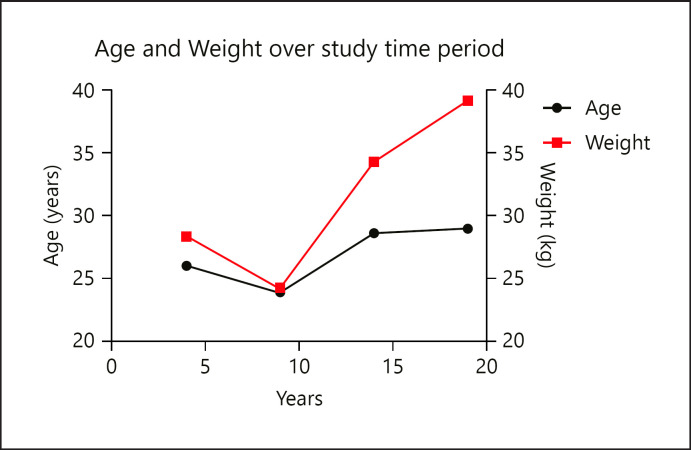
Average age and weight of all patients with paronychias over the 19-year study time period.

A total of 2,512 cases of paronychia were included in the final analysis, with a predominance of males (1,370, 54.5%). The average age was 27.6 years old (SD: 20.6) and the average weight was 33.8 kg (SD: 29.5) (Table 1). On multivariable linear regression including age, weight, and sex, both age (Estimate: 3.8, SE: 1.7, *p* = 0.03) and weight (Estimate: 9.5, SE: 1.2, *p* < 0.0001) significantly increased over time, while sex distribution remained unchanged (Fig. 1; Table 2). Of patients with recorded race, 644 (25.6%) were white and 718 (28.6%) were black (Table 1). The number of patients with paronychia who were identified as white or black decreased, with an increase in the number of patients with unknown race in 2013–2018, compared to 1999–2003 (Table 2). The most frequent site of paronychia for the hands and feet were the thumb (15.5%) and great toe (35.4%), respectively (Table 1). Overall, the proportion of thumb paronychias decreased from 19.9% in 1999–2003 to 11.0% in 2013–2018 (Table 2). All patients (100.0%) were treated in the emergency department and discharged or examined in the emergency department with no treatment required.

Over the study period, pediatric age and age across all cohorts increased (Table 2). The number of paronychia cases for pediatric patients ages 0–4 fell from 20.1% in 1999–2003 to 9.5% in 2013–2018, with relative increases in the number of patients ages 15–20 and over 20 years old. Across all age groups, the percentage of paronychia cases in patients ages 0–19 fell from 48.7% in 1999–2003 to 39.2% in 2013–2018, while the percentage in patients ages 20–39 increased from 21.4% to 31.4% across the same time period.

Manicuring tools were the most frequently cited causes of paronychia (776, 30.9%) (Table 3). For patients over the age of 21, 74.2% paronychias were due to manicuring tools, and only 12.6% were due to sports equipment (*p* < 0.0001). Manicuring (58.9%) and needle (55.5%) associated paronychias more commonly affected women, while door/drawer (56.8%), footwear (55.7%), sports equipment (85.3%), swimming pool (63.0%), knife (64.1%), and other (61.5%) associated paronychias were more frequent in men (*p* < 0.0001). Manicuring (25.9%), footwear (27.3%), knife (30.8%), and other (29.1%) associated paronychias were more common in whites, whereas door/drawer (40.2%), needle/nail (33.6%), sports equipment (51.6%), and swimming pool (50.0%) causes were more frequent in blacks (*p* < 0.0001). There were marked increases in manicuring tool associated paronychias and decreases in swimming pool associated paronychias over the four time periods (Table 2; *p* < 0.0001). Manicuring associated paronychias versus all other causes were more common in patients 21 and over, females, and patients of unknown race, with higher incidence in the latter two study periods of 2008–2013 and 2013–2018 (*p* < 0.0001, Table 3). There were no cases of paronychia caused by medications or irritants.

Paronychia site was compared between patients under 21 years and those aged 21 years and over (Table 4). Thumb paronychias were more common in those under 21, while index finger, middle finger, and ring finger injuries were all more common in those aged 21 and over (*p* = 0.0017). Patients aged 21 and over had greater frequencies of paronychias in all toes other than the fifth toe, and patients under 21 had a greater frequency of multiple toes being injured (*p* = 0.0105). Yearly numbers of paronychia cases in those over 21 increased over time (*p* < 0.0001).

## Discussion

Our cross-sectional analysis of 2,512 paronychia cases reported in the NEISS database from December 1999 to December 2018, is the largest study on paronychias to date. While we could not distinguish between acute, chronic, and chemotherapy-associated paronychias, the vast majority of paronychias were likely acute, since there were no cases of irritant or medication-associated paronychias. There were slightly greater proportions of male (54.5%) versus female (45.5%) patients diagnosed with paronychia. This finding is contrary to previous paronychia studies, reporting a strong female predominance, with one retrospective single-hospital study (*n* = 110) describing a population of 70.0% females and another prospective study in India (*n* = 80) reporting 82.5% females [15, 16]. This discrepancy may be due to the larger sample size and inclusion of likely predominantly acute paronychias due to a broad range of etiologies, in our study, including sports equipment and knives, which were more common in males than females. Of note, previous paronychia studies mainly focused on chronic paronychias [15, 16]. We noticed a large increase in both the number and percentage of paronychias due to manicuring tools over time, from 46 (5.9%) cases in 1999–2003 to 337 (43.4%) cases in 2013–2018, with the majority of these patients being female. All patients in our study were treated and discharged from the emergency department or did not need treatment. Acute paronychias can be drained and cultured by board-certified dermatologists and do not require the use of emergency services [17, 18]. Therefore, there is an unmet need to educate patients that dermatologists are experts in diagnosing and treating nail infections and that these can be easily managed in the outpatient setting. Some patients may have presented to emergency departments due to a paucity of dermatologists in those regions [19, 20], which is another challenge that needs to be addressed.

Unlike other more common nail diseases, such as onychomycosis, with robust clinical trial data [21], previous smaller paronychia studies lacked characterization of races. We found approximately equal proportions of white (25.6%) and black (28.6%) patients diagnosed with paronychias. We also observed a trend of race being reported less often over time in the NEISS database. Therefore, there is a significant deficit in race reporting in paronychia studies, highlighting the need for collection of this demographic information to better understand disease presentation.

In our study, the thumb (15.5%) and great toe (35.4%) were the most commonly involved upper and lower extremity digit paronychias, respectively. These findings match those of a retrospective chart review of 86 patients on the most common sites of extremity tendon and bone injuries [22, 23]. Similarly, in a prospective study on upper extremity acute paronychias (*n* = 31), the thumb and index finger of the dominant hand were most frequently affected [24, 25].

The age of patients diagnosed with paronychia increased over time for all age groups as well as pediatric patients. From 1999–2003 to 2013–2018, there was an approximately 10% decrease and a corresponding increase in paronychia cases in patients ages 0–19 and 20–39, respectively. During this time period, much of the decrease in pediatric paronychia cases was in the 0–4 age group, which may be due to better parental education on prevention of trauma and thumb-sucking behavior [26, 27]. Paronychias due to manicuring tools were approximately three times more common in patients over 21 and older (74.2%) versus those under 21 years old (25.8%) and nearly doubled (19.7%–37.2%) in incidence between 1999–2003 and 2013–2018. The increase in paronychia cases in patients ages 20–39 was likely due to the increased popularity in manicuring over time, with a 300% increase in both the number of nail salons and nail salon technicians nationally from 2000 to 2020 [28] and the global retail market for nail polish increasing from USD 3 billion in 2007 to USD 45 billion in 2012 [29].

Significant differences were seen in paronychia sites when stratified by age group. For the upper extremity, patients ages 21 and older experienced decreased rates of thumb paronychias and increased rates of paronychias in other digits, possibly due to a greater prevalence of manicuring in those over 21 (exposing digits other than the thumb to injury). While the thumb was the most frequently impacted finger in those under 21, the opposite trend was seen in the lower extremities, with lower percentages of great toe paronychias in those under 21 (32.8%) than those over 21 (38.4%). This finding is contrary to what was expected since congenital nail malformations would theoretically predispose patients to paronychias earlier in life [30]. Therefore, patients with congenital nail disorders, such as congenital malformation of the great toenail, may be more likely to be seen as outpatients rather than in emergency departments [31, 32].

Obesity has not previously been cited as a significant risk factor for the development of paronychia, but we observed a significant increase in average weight in patients with paronychias between 1999 and 2003 (28.3 kg) and 2013–2018 (39.2 kg). This rate of increase exceeded that of the general U.S. weight change rate over the same years, which increased from 25% to more than 30% [33]. Obesity is associated with increased infection rates [34]. For instance, in a cross-sectional study in the Netherlands of 8-year-olds (*n* = 3,960), children with BMI over 30 kg/m^2^ had fivefold greater rates of bronchitis and use of antibiotics compared to those with BMIs less than 30 kg/m^2^ [35]. While the reasons for this association are not clear, increased weight may lead to nail fold hypertrophy, susceptibility to onychocryptosis, and subsequent paronychias [36].

On multivariable linear regression analysis, both age and weight increased significantly over time, but sex remained unchanged. These findings suggest that both age and weight of patients with paronychias have independently increased over the study period and that the increase in weight was not solely due to the growing age of patients in our sample. Therefore, patients affected by paronychias were older and heavier over time.

Our study is subject to several limitations. The data in NEISS is retrospective and since reporting is by emergency department staff, some cases may have been missed or incorrectly classified as paronychia. Since our search algorithm searched for the keyword “paronychia”, if paronychia cases were described differently, we would have underestimated the true prevalence. This may account for the predominantly younger age of presentation of paronychia cases in our study, which may differ from prior clinical experience; however, this was the only way to ensure our study was limited to paronychias. Furthermore, as physicians not formally trained in nail disease may not fully understand the diagnosis of paronychia, the number of true paronychia cases in the sample remains unknown, especially given the lack of biopsy-related information. Characteristics, including race and digit affected, also had a majority of results recorded as “unknown” or “not otherwise specified”. We could not distinguish between acute, chronic, and chemotherapy-associated paronychias, or infecting organisms or treatments. Paronychia etiologies include a wide range of bacterial organisms, including *Staphylococcus aureus* and *Eiknella corrodens* (often associated with oral secretions through nail biting or thumb sucking), and nonbacterial organisms, like *Candida albicans* or herpes simplex [2, 17, 37]. These pathogens could not be distinguished in the NEISS database. Furthermore, the database lacked specific information on paronychia treatments, an important consideration that should be targeted in future studies.

## Conclusion

In our cross-sectional nationally representative analysis of 2,512 paronychia cases, the thumb and multiple toes or toes other than the great toe were most commonly affected in patients under 21 years of age, with the second, third, and fourth fingers and great toe more often involved in patients aged 21 and over. Patient age and weight independently increased over the 19-year study period, suggesting that weight is an important risk factor for the development of paronychia. There has been a sharp increase in paronychias due to manicuring tools in adults and decreased pediatric cases of paronychia, specifically in those 0–4 years old. Since no patients presenting with paronychias required hospital admission, we stress the need for increased public education that dermatologists are experts in managing nail disease, which can be done in the outpatient setting. We also highlight the need for improved reporting of race and treatments for paronychia patients for optimal prevention and management.

## Statement of Ethics

The NEISS database ethics statements can be found on the database's website. Subjects were coded in an anonymous fashion without the use of identifiers by emergency department staff nationwide, in accordance with the World Medical Association Declaration of Helsinki. Ethics approval and written informed consent were not required as per national guidelines.

## Conflict of Interest Statement

Dr. Lipner has served as a consultant for Orth-Dermatologics, Verrica, and Hoth Therapeutics.

## Funding Sources

There were no funding sources.

## Author Contributions

A.D.D. contributed to data analysis, manuscript preparation, and editing. Y.W. and C.C.N. contributed to idea curation, data curation, and data analysis. S.R.L. contributed to idea curation, manuscript preparation, and editing.

## Data Availability Statement

The primary dataset (National Electronic Injury Surveillance System) is available publicly through the US Consumer Product Safety Commission (https://www.cpsc.gov/Research–Statistics/NEISS-Injury-Data). The datasets generated and/or analyzed during the current study are available from the corresponding author on reasonable request.

## Figures and Tables

**Table 1:** Demographic characteristics of 2,512 patients diagnosed with paronychias

Characteristics	Mean (SD) or frequency (%)
Age, years	27.6 (20.6)
Sex	
Male	1,370 (54.5)
Female	1,142 (45.5)
Race	
White	644 (25.6)
Black	718 (28.6)
Other	276 (11.0)
Unknown	874 (34.8)
Weight, kg	33.8 (29.5)
Finger affected	
Thumb	228 (15.5)
2nd finger	125 (8.5)
3rd finger	181 (12.3)
4th finger	86 (5.9)
5th finger	19 (1.3)
Single finger, NOS	758 (51.6)
Multiple fingers, hand	71 (4.8)
N/A	1,044
Toe affected	
Great toe	284 (35.4)
2nd toe	10 (1.3)
3rd toe	11 (1.4)
4th toe	6 (0.8)
5th toe	11 (1.4)
Single toe, NOS	417 (51.9)
Multiple toes, foot	64 (8.0)
N/A	1,709

**Table 2:** Characteristics of patients diagnosed with paronychias over time

Characteristics	1999–2003 (*n* = 234) (9.3%)	2003–2008 (*n* = 436) (17.4%)	2008–2013 *(n* = 936) (37.3%)	2013–2018 *(n* = 906) (36.1%)	*p* value
Age					**<0.0001**
0–19 years	114 (48.7)	236 (54.1)	381 (40.7)	355 (39.2)	
20–39 years	50 (21.4)	92 (21.1)	269 (28.7)	284 (31.4)	
40–59 years	47 (20.1)	88 (20.2)	217 (23.2)	185 (20.4)	
60–79 years	21 (9.0)	18 (4.1)	54 (5.8)	73 (8.1)	
80+ years	2 (0.9)	2 (0.5)	15 (1.6)	9 (1.0)	
Pediatric versus adult age					**<0.0001**
0–4 years	47 (20.1)	86 (19.7)	120 (12.8)	86 (9.5)	
5–9 years	25 (10.7)	53 (12.2)	88 (9.4)	99 (10.9)	
10–14 years	29 (12.4)	54 (12.4)	88 (9.4)	99 (10.9)	
15–20 years	13 (5.6)	43 (9.9)	82 (8.8)	81 (8.9)	
Adult patients	120 (51.3)	200 (45.9)	555 (59.3)	551 (60.8)	
Sex					0.1175
Male	117 (50.0)	245 (56.2)	532 (56.8)	476 (52.5)	
Female	117 (50.0)	191 (43.8)	404 (43.2)	430 (47.5)	
Race					**<0.0001**
White	78 (33.3)	98 (22.5)	238 (25.4)	230 (25.4)	
Black	78 (33.3)	160 (36.7)	260 (27.8)	220 (24.3)	
Other	23 (9.8)	45 (10.3)	136 (14.5)	72 (8.0)	
Unknown	55 (23.5)	133 (30.5)	302 (32.3)	384 (42.4)	
Weight, kg	28.3 (SD: 25.3)	24.2 (SD: 24.1)	34.3 (SD: 29.3)	39.2 (SD: 31.8)	**<0.0001**
Finger affected					**0.0025**
Thumb	29 (19.9)	45 (17.7)	98 (17.5)	56 (11.0)	
2nd finger	11 (7.5)	17 (6.7)	49 (8.8)	48 (9.5)	
3rd finger	15 (10.3)	20 (7.9)	86 (15.4)	60 (11.8)	
4th finger	11 (7.5)	7 (2.8)	34 (6.1)	34 (6.7)	
5th finger	2 (1.4)	5 (2.0)	6 (1.1)	6 (1.2)	
Single finger, NOS	67 (45.9)	151 (59.5)	259 (46.3)	281 (56.3)	
Multiple fingers, hand	11 (7.5)	9 (3.5)	28 (5.0)	23 (4.5)	
Toe affected					**0.0152**
Great toe	22 (32.4)	43 (31.6)	130 (43.2)	89 (29.9)	
2nd toe	0 (0)	0 (0)	2 (0.7)	8 (2.7)	
3rd toe	1 (1.5)	2 (1.5)	6 (2.0)	2 (0.7)	
4th toe	0 (0)	3 (2.2)	2 (0.7)	1 (0.3)	
5th toe	1 (1.5)	1 (0.7)	5 (1.7)	4 (1.3)	
Single toe, NOS	42 (61.8)	75 (55.2)	132 (43.9)	168 (56.4)	
Multiple toes, foot	2 (2.9)	12 (8.8)	24 (8.0)	26 (8.7)	
Etiology					**<0.0001**
Manicure, pedicure, and make-up brushes and tools	46 (19.7)	86 (19.7)	307 (32.8)	337 (37.2)	
Doors and drawers	14 (6.0)	91 (20.9)	143 (15.3)	125 (13.8)	
Footwear	9 (3.9)	13 (3.0)	34 (3.6)	32 (3.5)	
Needles or nails	12 (5.1)	27 (6.2)	39 (4.2)	32 (3.5)	
Sports equipment	8 (3.4)	21 (4.8)	32 (3.4)	34 (3.8)	
Swimming pools	8 (3.4)	11 (2.5)	14 (1.5)	13 (1.4)	
Knives	4 (1.7)	6 (1.4)	12 (1.3)	17 (1.9)	
Other etiology	133 (56.8)	181 (41.5)	355 (37.9)	316 (34.9)	

**Table 3:** Comparison of characteristics between included etiologies

	Etiology									Manicure, pedicure, and make-up brushes and tools	All other products	*p* value
	manicure, pedicure, and make-up brushes and tools (*n =* 776) (30.9%)	doors or drawers (*n* = 373) (14.8%)	footwear (*n* = 88) (3.5%)	needles or nails (*n* = 110) (4.4%)	sports equipment (*n* = 95) (3.8%)	swimming pools (*n* = 46) (1.8%)	knives (*n* = 39) (1.6%)	other etiology (*n* = 985) (39.2%)	*p* value			
Age									**<0.0001**			**<0.0001**
Under 21 years	200 (25.8)	256 (68.6)	42 (47.7)	29 (26.4)	83 (87.4)	29 (63.0)	8 (20.5)	485 (49.2)		200 (25.8)	932 (53.7)	
21 years and over	576 (74.2)	117 (31.4)	46 (52.3)	81 (73.6)	12 (12.6)	17 (37.0)	31 (79.5)	500 (50.8)		576 (74.2)	804 (46.3)	
Sex									**<0.0001**			**<0.0001**
Male	319(41.1)	212 (56.8)	49 (55.7)	49 (44.6)	81 (85.3)	29 (63.0)	25 (64.1)	606 (61.5)		319 (41.1)	1,051 (60.5)	
Female	457 (58.9)	161 (43.2)	39 (44.3)	61 (55.5)	14 (14.7)	17 (37.0)	14 (35.9)	379 (38.5)		457 (58.9)	685 (39.5)	
Race									**<0.0001**			**<0.0001**
White	201 (25.9)	70 (18.8)	24 (27.3)	32 (29.1)	13 (13.7)	5 (10.9)	12 (30.8)	287 (29.1)		201 (25.9)	557 (32.1)	
Black	164 (21.1)	150 (40.2)	21 (23.9)	37 (33.6)	49 (51.6)	23 (50.0)	7 (18.0)	267 (27.1)		164 (21.1)	443 (25.5)	
Other	94 (12.11)	46 (12.3)	9 (10.2)	12 (10.9)	12 (12.6)	2 (4.4)	4 (10.3)	97 (9.9)		94 (12.11)	554 (31.9)	
Unknown	317 (40.9)	107 (28.7)	34 (38.6)	29 (26.4)	21 (22.1)	16 (34.8)	16 (41.0)	334 (33.9)		317 (40.9)	182 (10.5)	
Year									**<0.0001**			**<0.0001**
1999–2003	46 (5.9)	14 (3.8)	9 (10.2)	12 (10.9)	8 (8.4)	8 (17.4)	4 (10.3)	133 (13.5)		46 (5.9)	188 (10.8)	
2003–2008	86 (11.1)	91 (24.4)	13 (14.8)	27 (24.6)	21 (22.1)	11 (23.9)	6 (15.4)	181 (18.4)		86 (11.1)	350 (20.2)	
2008–2013	307 (39.6)	143 (38.3)	34 (38.6)	39 (35.5)	32 (33.7)	14 (30.4)	12 (30.8)	355 (36.0)		307 (39.6)	629 (36.2)	
2013–2018	337 (43.4)	125 (33.5)	32 (36.4)	32 (29.1)	34 (35.8)	13 (28.3)	17 (43.6)	316 (32.1)		337 (43.4)	569 (32.8)	

**Table 4:** Digit(s) affected by paronychia by age group

Characteristics	Under 21 years	21 years and over	*p* value
Finger affected			**0.0017**
Thumb	108 (17.1)	120 (14.3)	
2nd finger	41 (6.5)	84 (10.0)	
3rd finger	61 (9.7)	120 (14.3)	
4th finger	28 (4.4)	58 (6.9)	
5th finger	9 (1.4)	10 (1.2)	
Single finger, NOS	348 (55.2)	410 (48.9)	
Multiple fingers, hand	35 (5.6)	36 (4.3)	
Toe affected			**0.0105**
Great toe	143 (32.8)	141 (38.4)	
2nd toe	4 (0.9)	6 (1.6)	
3rd toe	3 (0.7)	8 (2.2)	
4th toe	1 (0.2)	5 (1.4)	
5th toe	7 (1.6)	4 (1.1)	
Single toe, NOS	233 (53.4)	184 (50.1)	
Multiple toes, foot	45 (10.3)	19 (5.2)	
Year			**<0.0001**
1999–2003	119 (10.5)	115 (8.3)	
2003–2008	242 (21.4)	194 (14.1)	
2008–2013	397 (35.1)	539 (39.1)	
2013–2018	374 (33.0)	532 (38.6)	
